# The Clinical Significance of Serum Biomarkers of the Intestinal Barrier in Systemic Sclerosis: A Cross-Sectional Study

**DOI:** 10.3390/jpm13040678

**Published:** 2023-04-18

**Authors:** Albert Stec, Magdalena Maciejewska, Michał Zaremba, Karolina Paralusz-Stec, Milena Michalska, Lidia Rudnicka, Mariusz Sikora

**Affiliations:** 1 Department of Dermatology, Medical University of Warsaw, Koszykowa 82A, 02-008 Warsaw, Poland; 2 Department of General, Vascular and Transplant Surgery, Medical University of Warsaw, Banacha 1a, 02-097 Warsaw, Poland; 3 National Institute of Geriatrics, Rheumatology and Rehabilitation, Spartańska 1, 02-637 Warsaw, Poland; drmariuszsikora@gmail.com

**Keywords:** systemic sclerosis, microbiota, gut–skin axis, dysbiosis, inflammation, immune-mediated inflammatory diseases, intestinal barrier, intestinal permeability

## Abstract

Systemic sclerosis (SSc) is an immune-mediated connective tissue disease. Recent studies reported differences in the composition of intestinal microbiota (dysbiosis) in patients with SSc compared to nonsclerodermic subjects. Dysbiosis may disrupt the intestinal barrier, which leads to immunological activation via microbial antigen and metabolite translocation. The study aimed to assess the differences in intestinal permeability between SSc patients and controls and to examine the correlation between intestinal permeability and complications of SSc. The study comprised 50 patients with SSc and 30 matched subjects. Serum intestinal permeability markers: intestinal fatty acid binding protein, claudin-3, and lipopolysaccharides (LPS) were determined using an enzyme-linked immunosorbent assay. SSc patients had a significantly increased concentration of LPS compared to control subjects (232.30 [149.00–347.70] versus 161.00 [83.92–252.20] pg/mL, *p* < 0.05). The patients with shorter SSc duration (≤6 years) had an increased concentration of LPS and claudin-3 compared to the subgroup with longer disease length: LPS (280.75 [167.30–403.40] versus 186.00 [98.12–275.90] pg/mL, *p* < 0.05), and claudin-3 (16.99 [12.41–39.59] versus 13.54 [10.29–15.47] ng/mL, *p* < 0.05). The patients with esophageal dysmotility had a decreased LPS level compared to those without this complication (188.05 [102.31–264.40] versus 283.95 [203.20–356.30] pg/mL, *p* < 0.05). Increased intestinal permeability in SSc may exacerbate the course of the disease and increase the risk of developing complications. Lower LPS levels in SSc might be a hallmark of esophageal dysmotility.

## 1. Introduction

Systemic sclerosis (SSc) is an immune-mediated connective tissue disease with a chronic, progressive course that causes multiorgan failure and the patient’s disability [1]. The disease is characterized by progressive fibrosis of skin and internal organs with concomitant impairment of microcirculation and persistent inflammation. The pathogenesis of systemic sclerosis is still poorly understood [1].

Accumulating evidence suggests that alterations in the composition of the gut microbiome, which is called dysbiosis, may play a role in the pathogenesis of systemic sclerosis [2,3,4] and other immune-mediated skin conditions such as psoriasis [5] and systemic lupus erythematosus [6]. Changes in the intestinal microbiota in the course of systemic sclerosis are characterized by an increased presence of bacteria of the genera *Fusobacterium*, *Desulfovibrio*, *Ruminococcus*, and *Lactobacillus* and a decreased presence of bacteria of the genus *Faecalibacterium* [2,3,4]. The concept of the gut-skin axis suggests a connection between changes in the intestinal microbiota and skin immunological responses [7]. However, the exact mechanism causing this crosstalk is unknown yet. One of the possible factors is the disruption of the intestinal barrier, which causes increased intestinal permeability. It can result in the translocation of intestinal luminal content, e.g., allergens, bacterial endotoxins, metabolites, or even whole bacterial cell components, into the circulation, which further initiates or exacerbates systemic inflammation [8,9].

Techniques that have been used so far for determining gut barrier integrity, such as histological examination of intestinal biopsies or oligosaccharide absorption assays, are complicated, time-consuming, and difficult to apply in routine clinical practice [10]. Alternatives for the mentioned methods include novel biomarkers, which can be assessed from the blood in routine blood collection. The biomarkers are intestinal fatty acid binding protein (IFABP), claudin-3, and lipopolysaccharides (LPS). IFABP is a protein that exclusively presents in the cytoplasm of enterocytes in the small intestine. In normal conditions, passage of this marker into circulation is minimal, whereas damage to the intestinal epithelium causes a marked increase of IFABP levels in the blood [11]. In turn, claudin-3 is a component of tight junctions between the enterocytes, and their disruption can be observed as an increase in the blood level of this marker [12]. LPS is a marker of the translocation of bacteria through the intestinal epithelium [9,10]. An increased concentration of this element indicates an extensive bacterial translocation and, additionally, it is a potent proinflammatory factor that may exacerbate inflammatory responses [9,13]. When measured together, the mentioned markers can reliably provide information about the state of the intestinal barrier.

The study aimed to investigate potential differences in intestinal permeability between the patients with systemic sclerosis and the matched controls with the use of serum intestinal permeability markers. The impact of various clinical factors on the concentration of the mentioned markers was also assessed.

## 2. Materials and Methods

### 2.1. Study Participants

The study comprised 50 adult patients diagnosed with systemic sclerosis who fulfilled the classification criteria of the American College of Rheumatology (ACR) and European League Against Rheumatism (EULAR) 2013. Those patients were recruited from the Department of Dermatology at the Medical University of Warsaw between January 2022 and June 2022.

To avoid potential bias the following exclusion criteria were used: acute or chronic gastrointestinal infection within the three months before the study, concomitant inflammatory bowel disease, any gastrointestinal surgery or unexplained weight loss within the six months before, intake of nonsteroidal anti-inflammatory drugs within the previous week, dietary restrictions, intake of antibiotics, probiotics or synbiotics within the previous 3 months, a history of malignancy, drug or alcohol abuse, chronic liver and pancreatic disease, estimated glomerular filtration rate (eGFR) of <60 mL/min/1.73 m^2^, pregnancy, and breastfeeding.

The control group consisted of 30 individuals who were matched for age, gender, and body mass index (BMI). Subjects in the control group met the same exclusion criteria.

### 2.2. Clinical Assessment

Detailed medical history was obtained from all of the participants. The severity of the disease was assessed with Valentini Disease Activity Score. The stage of skin involvement was analyzed with the modified Rodnan skin score (mRSS). To evaluate the gastrointestinal manifestation of systemic sclerosis a normal barium swallow was performed. In addition, high-resolution computed tomography (HRCT) and body plethysmography were performed to determine lung involvement. Besides, the cardiac manifestation of systemic sclerosis was assessed in echocardiography as well as by measuring N-terminal pro b-type natriuretic peptide (NT-proBNP).

### 2.3. Laboratory Assessment

The patients had the following tests performed: complete blood count, erythrocyte sedimentation rate, creatinine, and estimated glomerular filtration rate (calculated using the CDK-EPI equation). The venous blood samples were taken after a 12-h fast.

Antinuclear antibodies were evaluated by indirect immunofluorescence pattern on HEp-2 cells and detected by immunoblot analysis.

The samples were tested by use of commercially available ELISA kits: I-FABP: R&D Systems, Inc., Minneapolis, MN, USA (assay range: 15.6 pg/mL–1000 pg/mL); LPS: CUSABIO, Wuhan, China (assay range: 6.25 pg/mL–400 pg/mL); and CLDN3: Wuhan Fine Biotech Co., Wuhan, China (assay range: 0.313 ng/mL–20 ng/mL) in order to quantify the serum concentration of permeability markers. The concentrations of target proteins in the test samples were estimated based on the previous studies, and a proper dilution factor was selected to make the diluted target proteins’ concentration fall within the optimal detection range of the kit. The concentrations read from the standard curve were multiplied by the dilution factor. The samples for these analyses were collected according to the manufacturers’ instructions: blood samples were collected from the peripheral vein after a 12-h fasting period and placed in the serum separator tubes. After 2 h of clotting, the samples were centrifuged for 20 min at 1000× *g*. Serum aliquots obtained by centrifugation were stored at −80 °C until further analysis. All of the measurements were performed in accordance with the manufacturers’ instructions. Additionally, they were assessed in duplicate and the means were utilized to further analysis. For the ELISA analyses mentioned above, intra-assay coefficients of variation were below 8% and inter-assay coefficients of variation were below 11%.

### 2.4. Statistical Analysis

The statistical software STATISTICA 13.1 was used for all calculations (StatSoft, Krakow, Poland). The Shapiro-Wilk test was applied so as to determine if a continuous variable follows a normal distribution. The mean standard deviation (SD) was used to represent normally distributed data, whereas the median and interquartile range were utilized to describe non-normally distributed variables (IQR). A chi-square test (with the Yates correction for small groups [n < 10]) was used to compare categorical data that were presented as counts and percentages. The Student’s *t*-test was chosen to assess the continuous variables with a parametric distribution, whereas the Mann-Whitney U test was selected in order to assess continuous variables with a nonparametric distribution. Besides, Spearman’s rank correlation coefficient was applied to evaluate potential correlations between two continuous variables. The *p*-value of <0.05 was considered statistically significant.

### 2.5. Ethics

The study was approved by the Regional Bioethical Committee at the Medical University of Warsaw. Written informed consent was obtained from all of the participants. The study was carried out in accordance with the Declaration of Helsinki, and it also gained the approval of the local bioethical committee (approval code: KB136/2021; Bioethics Committee at the Medical University of Warsaw, Warsaw, Poland).

## 3. Results

### 3.1. Patients’ Characteristics

The study included 50 patients with systemic sclerosis and 30 age-, sex-, and BMI-matched control individuals. According to the design of the study, there was no difference between the two participant groups in terms of age, sex distribution, or BMI. Table 1 provides an overview of the key demographic, clinical, laboratory, and serological characteristics of individuals with SSc and the control group.

### 3.2. Markers of Intestinal Permeability in Systemic Sclerosis

**Figure 1:**
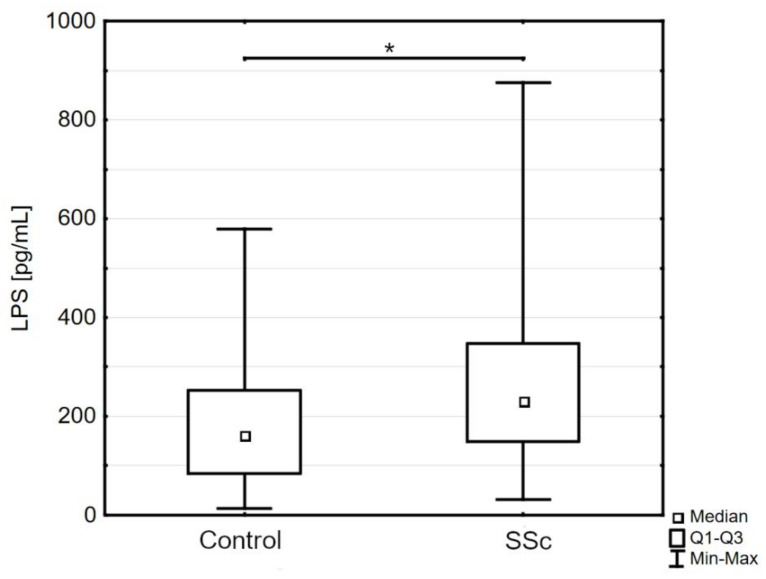
The serum concentration of lipopolysaccharides (LPS) in patients with systemic sclerosis compared to controls; SSc–systemic sclerosis; Q1—the first quartile; Q3—the third quartile; Min—the lowest value; Max—the highest value (* *p* < 0.05).

In patients with systemic sclerosis, we found a significantly higher concentration of LPS compared to control subjects (232.30 pg/mL [149.00–347.70] versus 161.00 pg/mL [83.92–252.20], *p* < 0.05; Figure 1). The differences between SSc and control groups in other markers of intestinal permeability, i.e., IFABP and claudin-3, were not statistically significant: IFABP (1505.0 pg/mL [1108.0–1865.0] versus 1598.0 pg/mL [921.8–1835.0], *p* = 0.45); claudin-3 (14.44 ng/mL [11.85–94.53] versus 15.22 ng/mL [11.83–23.11], *p* = 0.63).

### 3.3. Markers of Intestinal Permeability in Subgroups of Disease Duration

**Figure 2:**
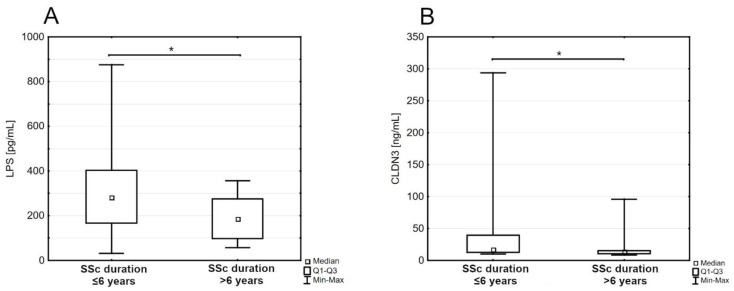
The serum concentration of lipopolysaccharides (LPS) (**A**) and claudin-3 (CLDN3) (**B**) in patients’ subgroups with different durations of systemic sclerosis (SSc); Q1—the first quartile; Q3—the third quartile; Min—the lowest value; Max—the highest value (* *p* < 0.05).

Further analysis revealed a significant negative correlation between claudin-3 and disease duration (rho = −0.31, *p* < 0.05). Because of this fact, the patients’ group has been split in two by the median disease duration. A comparison of levels of intestinal permeability markers between these two groups revealed a significantly higher concentration of LPS and claudin-3 in the group with a shorter duration of SSc than the group with a longer duration: LPS (280.75 pg/mL [167.30–403.40] versus 186.00 pg/mL [98.12–275.90], *p* < 0.05; Figure 2A); claudin-3 (16.99 ng/mL [12.41–39.59] versus 13.54 ng/mL [10.29–15.47], *p* < 0.05; Figure 2B). Detailed characteristics of both groups are provided in Table 2.

### 3.4. Lipopolysaccharides (LPS) in Specific Comorbidities of Systemic Sclerosis

**Figure 3:**
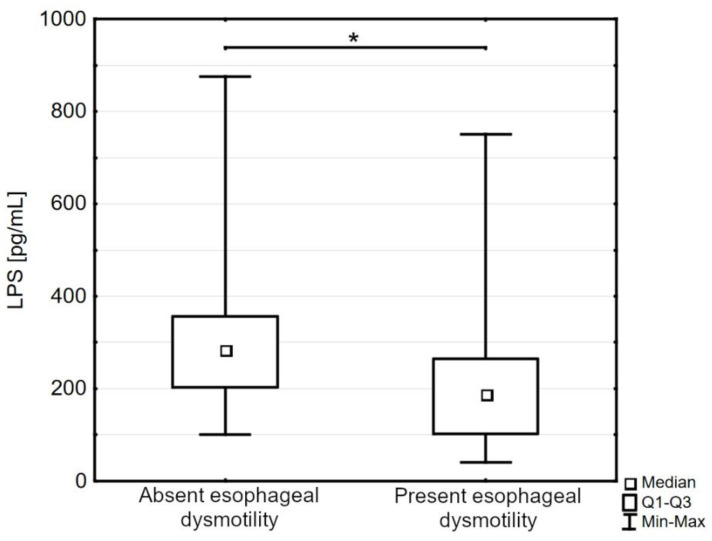
The serum concentration of lipopolysaccharides (LPS) in the patients with systemic sclerosis and absent esophageal dysmotility compared to the subgroup with present esophageal dysmotility; Q1—the first quartile; Q3—the third quartile; Min—the lowest value; Max—the highest value (* *p* < 0.05).

The patients with esophageal dysmotility were characterized by a significantly decreased level of LPS compared to the patients without this complication (188.05 pg/mL [102.31–264.40] versus 283.95 pg/mL [203.20–356.30], *p* < 0.05; Figure 3).

**Figure 4:**
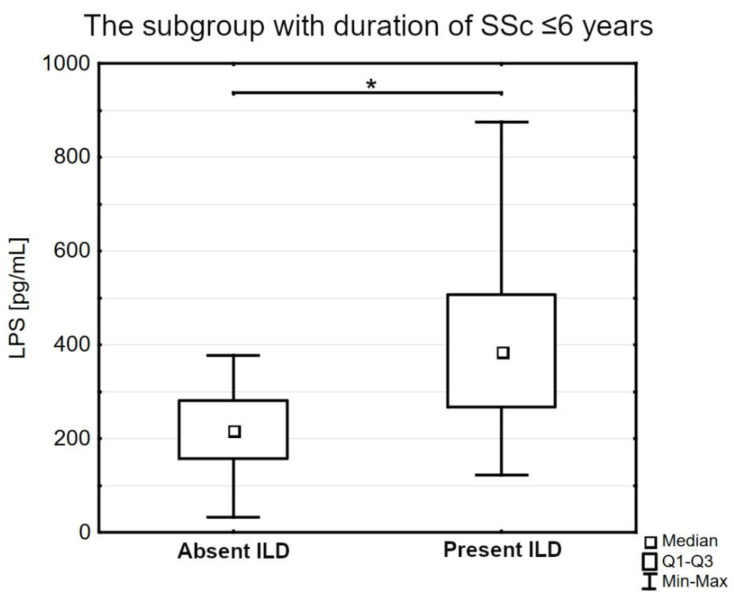
The serum concentration of lipopolysaccharides (LPS) in the patients’ subgroup with a shorter duration of the disease with or without concomitant interstitial lung disease (ILD); Q1—the first quartile; Q3—the third quartile; Min—the lowest value; Max—the highest value (* *p* < 0.05).

In the subgroup with a shorter duration of SSc and concomitant interstitial lung disease, we observed an increased level of LPS compared to the subgroup with a shorter duration of SSc and absent ILD (385.55 pg/mL [266.90–506.50] versus 217.75 pg/mL [157.25–280.75], *p* < 0.05; Figure 4).

We did not observe any relevant correlations between the markers themselves or between markers and left ventricle ejection fraction, NT-proBNP, DLCO, or characteristics of systemic sclerosis (i.e., disease activity index, modified Rodnan Skin score, disease subtype, antibody profile).

## 4. Discussion

Intestinal barrier integrity has recently been the focus of extensive studies about the interactions between the gastrointestinal tract and general homeostasis. The findings of our research are consistent with the previous studies and confirm that disruption of the intestinal barrier is a feature of the early stage of systemic sclerosis [14,15]. The assessment of intestinal permeability in the mentioned studies was based on sugar permeability tests and we observed similar results using serum protein markers. Furthermore, our findings seem to prove the hypothesis proposed in these studies regarding the translocation of pro-inflammatory antigens from the intestinal lumen induced by increased intestinal permeability [14,15].

The obtained findings could reflect the natural course of systemic sclerosis. Depending on the study, the highest disease activity and rate of progression occur between the 3rd and 5th years of the disease, and after this stage, the disease often slows down or even slightly reverses, as in the case of skin sclerosis [16,17]. Consistently, our study implies that in the early period of SSc, the intestinal barrier is damaged, which results in increased gut permeability and translocation of bacterial lipopolysaccharides. The observed results could be the consequence of abnormalities in the ultrastructure of the small intestinal mucosa found in progressive systemic sclerosis, mainly dilated intraepithelial spaces [18]. Since claudin-3 is an element of tight junctions, widened spaces between enterocytes suggest a disruption of these components, which could be the cause of an increased concentration of claudin-3 and LPS in patients with SSc [10]. The lack of differences in IFABP concentration between patients and controls may be due to the absence of pathology in enterocytes since IFABP is a protein that exclusively presents in the cytoplasm of these cells [10,18].

Lipopolysaccharides are well-recognized pro-inflammatory factors, and an increased serum LPS concentration in patients with early systemic sclerosis, which was observed in our study, may have a detrimental impact on the further course of the disease. Recent publications have reported that TLR4, which is a receptor for LPS among others, can be substantially involved in the pathogenesis of SSc [19]. Skin and lung biopsies from SSc patients are characterized by an increased presence of TLR4 on fibroblasts compared to nonsclerodermic subjects [20]. Moreover, in vitro studies revealed that LPS can stimulate the expression of extracellular matrix genes, especially collagen, in skin fibroblasts and markedly increase their capacity to initiate a profibrotic response when challenged with TGF-β1, a cytokine known as a major profibrotic trigger in SSc [20,21]. Furthermore, LPS can induce the transdifferentiation of skin fibroblasts into myofibroblasts and promote profibrotic gene expression, which is particularly important in the case of systemic sclerosis, in which myofibroblasts are responsible for organ fibrosis [20,22,23]. LPS is known for its ability to induce lung inflammation and fibrosis, and an LPS-treated mouse is a model for acute lung injury [24]. However, LPS in this model is administered at supraphysiological concentrations [24]. The data on the effects of prolonged exposition to lower doses on lung physiology remain elusive. Despite this fact, LPS was found to exacerbate coexisting interstitial lung disease in the mouse model [25]. Moreover, in patients with systemic sclerosis and concomitant intestinal lung disease, compared to the control group, blood monocytes were found to secrete significantly higher amounts of IL-6 after exposure to LPS [26]. IL-6 levels are elevated in the sera of SSc patients and have been shown to be strongly associated with the severity of skin thickening and disease progression in interstitial lung disease [27,28]. In this context, an approach based on lowering the exposure to LPS could be a potential therapeutic option in systemic sclerosis.

Observed lower in the subgroup with a longer disease duration than in the subgroup with a shorter disease duration, the concentration of permeability markers may be an effect of coexisting disturbances in intestinal absorption due to fibrosis. Impaired absorption of saccharides, i.e., lactose and fructose, was found to be often present in patients with systemic sclerosis [29,30]. Furthermore, the present malabsorption of the mentioned saccharides was associated with increased gastrointestinal symptoms [29,30]. Malabsorption can be a result of multifactorial pathology of the affected intestinal wall, which includes fibrosis of the arterial tunica intima in the submucosal arteries of the small bowel, damage to the enteric nervous system, and marked fibrosis of the circular layer of the muscularis propria [31,32]. Available diagnostic methods for the involvement of the gastrointestinal tract are limited, complex, and expensive [31]. Since we observed a significantly lower LPS concentration in the patients with esophageal involvement compared to patients with normal esophageal motility, it could be hypothesized that the lower translocation of LPS in those patients may be due to decreased intestinal absorption of LPS. Further studies are needed to evaluate this relationship and its clinical significance.

As mentioned, substances from the intestinal lumen, such as LPS, can reach the circulation and exert proinflammatory effects, which can potentially accelerate disease progression. Many prognostic factors have been identified as a consequence of the complicated pathophysiology of SSc, and the presence and interplay between them might reflect the course of the disease [33]. It is possible that increased intestinal permeability is one of the causes exacerbating the natural course of systemic sclerosis, and a further decrease in permeability markers could be an effect of developing fibrosis of the gastrointestinal tract. However, due to the limitations of our study, including the relatively small sample size, single-center type, and cross-sectional character, the conclusions on the impact on disease progression are limited. To exactly assess the pathogenetic effects of a disrupted gut barrier on the course of SSc, prospective, multicenter studies are needed. The prospective assessment of intestinal permeability and exposition to LPS at different stages of the disease seem to be particularly valuable.

Understanding the mechanisms of how dysbiotic microbiota and a disrupted intestinal barrier may influence the course of systemic sclerosis seems valuable in clinical practice. Hypothetically, measurement of the intestinal barrier markers may help to stratify patients into different prognostic categories, which could help optimize treatment in the early stages of the disease. An approach based on the modification of the gut microbiota could break the vicious circle driven by inflammation induced by increased translocation of bacterial elements. It could lead to mitigating the progression of the disease, especially in the early stages of the disease when the complications are not developed. Available techniques of modulation of microbiota include probiotics, prebiotics, and fecal microbiota transplantation. These methods have been intensively studied in a number of dermatological conditions and are known to improve the gut barrier [34,35,36]. An improvement of the intestinal barrier by the mentioned interventions leads to a decrease in intestinal permeability and can be an effect of elevated expression of proteins of tight junction complexes in intestinal epithelial cells, stimulation of the proliferation of intestinal epithelial cells, and an increased secretion of mucins, which protect enterocytes from stressors [34,35,36]. Such attempts were also made in systemic sclerosis and were associated mainly with the improvement of gastrointestinal symptoms [37,38,39,40,41]. One study reported the immunomodulatory effect of a probiotic mixture manifested as a significant decrease in the proportion of Th17 cells compared with placebo [38]. It is worth noting that the administration of probiotics as well as fecal microbiota transplantation exhibited an excellent safety profile, and the only noticed adverse events were mild and transient, i.e., diarrhea, bloating, and abdominal pain [37,38,39,40,41]. However, there is still little information available on the most appropriate probiotic strain composition and donor matching for fecal microbiota transplantation. Measurements of intestinal permeability markers in such trials could be a marker of intervention outcome.

## 5. Conclusions

Our cross-sectional study revealed that the patients with systemic sclerosis are characterized by increased LPS serum concentration compared to the control subjects. Furthermore, biomarkers of intestinal barrier permeability, LPS and claudin-3, are elevated in the patients with a shorter duration of the disease compared to the subgroup with a longer duration of the disease. Additionally, in the subgroup with a shorter duration of SSc, we observed increased LPS concentration in patients with concomitant interstitial lung disease compared to the patients with the absence of this complication. Concomitant esophageal dysmotility was associated with a decrease in LPS in the patients with SSc. The study highlighted the importance of disease duration in gut barrier research. On account of the potent proinflammatory and profibrotic properties of LPS, increased circulating LPS may be an exacerbating factor in the early stage of the disease, whereas the subsequent decrease of LPS may be due to the development of gastrointestinal disturbances. However, to exactly determine the impact of LPS in systemic sclerosis, prospective studies are needed to demonstrate the effect of prolonged exposition to LPS. The modulation of the gut barrier may represent a new therapeutic approach for systemic sclerosis.

## Figures and Tables

**Table 1:** The characteristics of the control group and individuals with SSc.

	Systemic Sclerosis (n = 50)	Control (n = 30)	*p*-Value
General characteristics			
Age, years	57 [48–65]	54 [49–59]	0.22
Sex, women, n (%)	42 (84.00%)	25 (83.33%)	0.94
Body mass index, kg/m^2^	23.56 [21.31–27.44]	25.29 [22.35–26.25]	0.67
Characteristics of systemic sclerosis			
Modified Rodnan skin score	4 [2–9]	-	-
Limited cutaneous systemic sclerosis, n (%)	27 (54%)	-	-
Diffuse cutaneous systemic sclerosis, n (%)	23 (46%)	-	-
Systemic sclerosis duration, years	6 [4–13]	-	-
Autoantibody positivity			
Anticentromere (ACA), n (%)	23 (46%)	-	-
Antitopoisomerase I (ATA), n (%)	19 (38%)	-	-
Anti-RNA polymerase III, n (%)	5 (10%)	-	-
Current treatment			
Methotrexate, n (%)	12 (24%)	-	-
Mycophenolate mofetil, n (%)	14 (28%)	-	-
Calcium channel blockers, n (%)	9 (18%)	-	-
Sildenafil, n (%)	25 (50%)	-	-
Sulodexide, n (%)	29 (58%)	-	-
Prostaglandins, n (%)	45 (90%)	-	-
Pentoxifylline, n (%)	4 (8%)	-	-

**Table 2:** The characteristics of the patients with shorter (≤6 years) and longer (>6 years) durations of systemic sclerosis.

	Shorter Duration of the Disease (≤6 Years; n = 28)	Longer Duration of the Disease (>6 years; n = 22)	*p*-Value
General characteristics			
Age, years	55.9 ± 11.7	57.0 ± 12.3	0.70
Sex, women, n (%)	21 (75.0%)	21 (95.5%)	0.12
Body mass index, kg/m^2^	23.1 [21.1–27.5]	23.8 [21.9–27.4]	0.70
Characteristics of systemic sclerosis			
Limited cutaneous systemic sclerosis, n (%)	16 (57.1%)	11 (50.0%)	0.62
Diffuse cutaneous systemic sclerosis, n (%)	12 (42.9%)	11 (50.0%)	0.62
Modified Rodnan skin score	4 [2–6]	4.5 [2–9]	0.41
Interstitial lung disease, n (%)	14 (50.0%)	19 (86.4%)	**0.02**
Diffusing capacity of the lungs for carbon monoxide (DLCO), %	71.14 ± 21.08	72.96 ± 13.62	0.62
Left ventricular ejection fraction (LVEF), %	65 [60–67]	65 [60–65]	0.99
Esophageal dysmotility, n (%)	13 (46.4%)	14 (63.6%)	0.55
Autoantibody positivity			
Anticentromere (ACA), n (%)	16 (57.1%)	7 (31.8%)	0.13
Antitopoisomerase I (ATA), n (%)	9 (32.1%)	10 (45.5%)	0.50
Anti-RNA polymerase III, n (%)	3 (10.7%)	2 (9.1%)	0.78
Intestinal barrier parameters			
Intestinal fatty acid binding protein (IFABP), pg/mL	1375.0 [1060.0–1828.5]	1534.0 [1108.0–1885.0]	0.61
Claudin-3 (CLDN3), ng/mL	16.99 [12.41–39.59]	13.54 [10.29–15.47]	**0.02**
Lipopolysaccharides (LPS), pg/mL	280.75 [167.30–403.40]	186.00 [98.12–275.90]	**0.02**
Laboratory parameters			
Erythrocyte sedimentation rate, mm/h	13.0 [6.0–21.0]	8.5 [7.0–12.0]	0.41
Estimated glomerular filtration rate (eGFR), mL/min./1.73 m^2^	87.41 ± 19.49	79.19 ± 18.28	0.15
N-terminal pro b-type natriuretic peptide (NT-proBNP), pg/mL	126 [74–230]	145 [64.5–206.5]	0.90

## Data Availability

The data that support the findings of this study are available from the corresponding author upon reasonable request.

## References

[1] Denton C.P., Khanna D. (2017). Systemic sclerosis. Lancet.

[2] Volkmann E.R., Hoffmann-Vold A.M., Chang Y.L., Jacobs J.P., Tillisch K., Mayer E.A., Clements P.J., Hov J.R., Kummen M., Midtvedt Ø. (2017). Systemic sclerosis is associated with specific alterations in gastrointestinal microbiota in two independent cohorts. BMJ Open Gastroenterol..

[3] Andreasson K., Lee S.M., Lagishetty V., Wu M., Howlett N., English J., Hesselstrand R., Clements P.J., Jacobs J.P., Volkmann E.R. (2022). Disease Features and Gastrointestinal Microbial Composition in Patients with Systemic Sclerosis from Two Independent Cohorts. ACR Open Rheumatol..

[4] Kim S., Park H.J., Lee S.I. (2022). The Microbiome in Systemic Sclerosis: Pathophysiology and Therapeutic Potential. Int. J. Mol. Sci..

[5] Sikora M., Stec A., Chrabaszcz M., Knot A., Waskiel-Burnat A., Rakowska A., Olszewska M., Rudnicka L. (2020). Gut Microbiome in Psoriasis: An Updated Review. Pathogens.

[6] Pan Q., Guo F., Huang Y., Li A., Chen S., Chen J., Liu H.F., Pan Q. (2021). Gut Microbiota Dysbiosis in Systemic Lupus Erythematosus: Novel Insights into Mechanisms and Promising Therapeutic Strategies. Front. Immunol..

[7] De Pessemier B., Grine L., Debaere M., Maes A., Paetzold B., Callewaert C. (2021). Gut-Skin Axis: Current Knowledge of the Interrelationship between Microbial Dysbiosis and Skin Conditions. Microorganisms.

[8] Camilleri M. (2019). Leaky gut: Mechanisms, measurement and clinical implications in humans. Gut.

[9] Ghosh S.S., Wang J., Yannie P.J., Ghosh S. (2020). Intestinal Barrier Dysfunction, LPS Translocation, and Disease Development. J. Endocr. Soc..

[10] Vanuytsel T., Tack J., Farre R. (2021). The Role of Intestinal Permeability in Gastrointestinal Disorders and Current Methods of Evaluation. Front. Nutr..

[11] Gajda A.M., Storch J. (2015). Enterocyte fatty acid-binding proteins (FABPs): Different functions of liver and intestinal FABPs in the intestine. Prostaglandins Leukot. Essent. Fat. Acids.

[12] Barmeyer C., Fromm M., Schulzke J.D. (2017). Active and passive involvement of claudins in the pathophysiology of intestinal inflammatory diseases. Pflug. Arch.

[13] Page M.J., Kell D.B., Pretorius E. (2022). The Role of Lipopolysaccharide-Induced Cell Signalling in Chronic Inflammation. Chronic Stress.

[14] Caserta L., de Magistris L., Secondulfo M., Caravelli G., Riegler G., Cuomo G., D’Angelo S., Naclerio C., Valentini G., Carratù R. (2003). Assessment of intestinal permeability and orocecal transit time in patients with systemic sclerosis: Analysis of relationships with epidemiologic and clinical parameters. Rheumatol. Int..

[15] Catanoso M., Lo Gullo R., Giofré M.R., Pallio S., Tortora A., Lo Presti M., Frisina N., Bagnato G., Fries W. (2001). Gastro-intestinal permeability is increased in patients with limited systemic sclerosis. Scand. J. Rheumatol..

[16] Medsger T.A. (2003). Natural history of systemic sclerosis and the assessment of disease activity, severity, functional status, and psychologic well-being. Rheum. Dis. Clin. North Am..

[17] Yanaba K. (2016). Strategy for treatment of fibrosis in systemic sclerosis: Present and future. J. Dermatol..

[18] Hendel L., Kobayasi T., Petri M. (1987). Ultrastructure of the small intestinal mucosa in progressive systemic sclerosis (PSS). Acta Pathol. Microbiol. Immunol. Scand A.

[19] O’Reilly S. (2023). Toll-like receptor triggering in systemic sclerosis: Time to target. Rheumatology.

[20] Bhattacharyya S., Kelley K., Melichian D.S., Tamaki Z., Fang F., Su Y., Feng G., Pope R.M., Budinger G.R., Mutlu G.M. (2013). Toll-like receptor 4 signaling augments transforming growth factor-beta responses: A novel mechanism for maintaining and amplifying fibrosis in scleroderma. Am. J. Pathol..

[21] Li X.P., Liu P., Li Y.F., Zhang G.L., Zeng D.S., Liu D.L. (2019). LPS induces activation of the TLR4 pathway in fibroblasts and promotes skin scar formation through collagen I and TGF-β in skin lesions. Int. J. Clin. Exp. Pathol..

[22] Cutolo M., Sulli A., Smith V. (2010). Assessing microvascular changes in systemic sclerosis diagnosis and management. Nat. Rev. Rheumatol..

[23] Zhan S., Li N., Liu C., Mao R., Wu D., Li T., Chen M., Zhuang X., Zeng Z. (2021). Intestinal Fibrosis and Gut Microbiota: Clues From Other Organs. Front. Microbiol..

[24] Domscheit H., Hegeman M.A., Carvalho N., Spieth P.M. (2020). Molecular Dynamics of Lipopolysaccharide-Induced Lung Injury in Rodents. Front. Physiol..

[25] Kimura T., Nojiri T., Hosoda H., Shintani Y., Inoue M., Miyazato M., Okumura M., Kangawa K. (2015). Exacerbation of bleomycin-induced injury by lipopolysaccharide in mice: Establishment of a mouse model for acute exacerbation of interstitial lung diseases. Eur. J. Cardiothorac. Surg..

[26] Crestani B., Seta N., De Bandt M., Soler P., Rolland C., Dehoux M., Boutten A., Dombret M.C., Palazzo E., Kahn M.F. (1994). Interleukin 6 secretion by monocytes and alveolar macrophages in systemic sclerosis with lung involvement. Am. J. Respir. Crit. Care Med..

[27] Cardoneanu A., Burlui A.M., Macovei L.A., Bratoiu I., Richter P., Rezus E. (2022). Targeting Systemic Sclerosis from Pathogenic Mechanisms to Clinical Manifestations: Why IL-6?. Biomedicines.

[28] Kawaguchi Y. (2017). Contribution of Interleukin-6 to the Pathogenesis of Systemic Sclerosis. J. Scleroderma Relat. Disord..

[29] Marie I., Leroi A.M., Gourcerol G., Levesque H., Menard J.F., Ducrotte P. (2015). Fructose Malabsorption in Systemic Sclerosis. Medicine.

[30] Marie I., Leroi A.M., Gourcerol G., Levesque H., Menard J.F., Ducrotte P. (2016). Lactose malabsorption in systemic sclerosis. Aliment. Pharmacol. Ther..

[31] McMahan Z.H., Kulkarni S., Chen J., Chen J.Z., Xavier R.J., Pasricha P.J., Khanna D. (2023). Systemic sclerosis gastrointestinal dysmotility: Risk factors, pathophysiology, diagnosis and management. Nat. Rev. Rheumatol..

[32] den Braber-Ymker M., Vonk M.C., Grunberg K., Lammens M., Nagtegaal I.D. (2021). Intestinal hypomotility in systemic sclerosis: A histological study into the sequence of events. Clin. Rheumatol..

[33] Pokeerbux M.R., Giovannelli J., Dauchet L., Mouthon L., Agard C., Lega J.C., Allanore Y., Jego P., Bienvenu B., Berthier S. (2019). Survival and prognosis factors in systemic sclerosis: Data of a French multicenter cohort, systematic review, and meta-analysis of the literature. Arthritis Res. Ther..

[34] Stec A., Sikora M., Maciejewska M., Paralusz-Stec K., Michalska M., Sikorska E., Rudnicka L. (2023). Bacterial Metabolites: A Link between Gut Microbiota and Dermatological Diseases. Int. J. Mol. Sci..

[35] Gou H.Z., Zhang Y.L., Ren L.F., Li Z.J., Zhang L. (2022). How do intestinal probiotics restore the intestinal barrier?. Front. Microbiol..

[36] Fortea M., Albert-Bayo M., Abril-Gil M., Ganda Mall J.P., Serra-Ruiz X., Henao-Paez A., Exposito E., Gonzalez-Castro A.M., Guagnozzi D., Lobo B. (2021). Present and Future Therapeutic Approaches to Barrier Dysfunction. Front. Nutr..

[37] Fretheim H., Chung B.K., Didriksen H., Baekkevold E.S., Midtvedt O., Brunborg C., Holm K., Valeur J., Tennoe A.H., Garen T. (2020). Fecal microbiota transplantation in systemic sclerosis: A double-blind, placebo-controlled randomized pilot trial. PLoS ONE.

[38] Marighela T.F., Arismendi M.I., Marvulle V., Brunialti M.K.C., Salomao R., Kayser C. (2019). Effect of probiotics on gastrointestinal symptoms and immune parameters in systemic sclerosis: A randomized placebo-controlled trial. Rheumatology.

[39] Low A.H.L., Teng G.G., Pettersson S., de Sessions P.F., Ho E.X.P., Fan Q., Chu C.W., Law A.H.N., Santosa A., Lim A.Y.N. (2019). A double-blind randomized placebo-controlled trial of probiotics in systemic sclerosis associated gastrointestinal disease. Semin. Arthritis Rheum..

[40] Strahm N., Didriksen H., Fretheim H., Molberg O., Midtvedt O., Farstad I.N., Midtvedt T., Lundin K.E.A., Aabakken L., Blyszczuk P. (2023). Effects of faecal microbiota transplantation on small intestinal mucosa in systemic sclerosis. Rheumatology.

[41] Frech T.M., Khanna D., Maranian P., Frech E.J., Sawitzke A.D., Murtaugh M.A. (2011). Probiotics for the treatment of systemic sclerosis-associated gastrointestinal bloating/distention. Clin. Exp. Rheumatol..

